# Critical Roles of the Circadian Transcription Factor *BMAL1* in Reproductive Endocrinology and Fertility

**DOI:** 10.3389/fendo.2022.818272

**Published:** 2022-03-02

**Authors:** Yin Jiang, Shiping Li, Wenming Xu, Junjie Ying, Yi Qu, Xiaohui Jiang, Ayuan Zhang, Yan Yue, Ruixi Zhou, Tiechao Ruan, Jinhui Li, Dezhi Mu

**Affiliations:** ^1^ Department of Pediatrics, West China Second University Hospital, Sichuan University, Chengdu, China; ^2^ Key Laboratory of Birth Defects and Related Diseases of Women and Children, Ministry of Education, Sichuan University, Chengdu, China; ^3^ Reproductive Endocrinology and Regulation Laboratory, West China Second University Hospital, Sichuan University, Chengdu, China; ^4^ Department of Andrology/Sichuan Human Sperm Bank, West China Second University Hospital, Sichuan University, Chengdu, China

**Keywords:** circadian gene *BMAL1*, reproductive endocrine disorder, fertility, circadian rhythm, reproduction

## Abstract

Brain and muscle aryl-hydrocarbon receptor nuclear translocator like protein1 (*BMAL1*), a core component of circadian oscillation, is involved in many physiological activities. Increasing evidence has demonstrated the essential role of *BMAL1* in reproductive physiology. For instance, *BMAL1*-knockout (KO) mice were infertile, with impaired reproductive organs and gametes. Additionally, in *BMAL1*-KO mice, hormone secretion and signaling of hypothalamus-pituitary-gonadal (H-P-G) hormones were also disrupted, indicating that H-P-G axis was impaired in *BMAL1*-KO mice. Moreover, both *BMAL1*-KO mice and BMAL1-knockdown by small interfering RNA (siRNA) *in vitro* cultured steroidogenic cells showed that *BMAL1* was associated with gonadal steroidogenesis and expression of related genes. Importantly, *BMAL1* also participates in pathogenesis of human reproductive diseases. In this review, we elaborate on the impaired reproduction of BMAL1-KO mice including the reproductive organs, reproductive endocrine hormones, and reproductive processes, highlighting the vital role of *BMAL1* in fertility and reproductive endocrinology.

## Introduction

Circadian rhythms play essential roles in various physiological processes as well as the development of organisms, which are now known to be largely controlled by a group of transcription factor genes. In mammals, such molecular clock is expressed and operational in most cells of the body (1). The central clock in the SCN (suprachiasmatic nucleus) is the master circadian oscillator that synchronizes the peripheral clocks (2, 3). The circadian clock transcription factor, *BMAL1* (brain and muscle aryl hydrocarbon receptor nuclear translocator like protein 1), also known as ARNTL1 or MOP3, which is expressed in most tissues especially in endocrine tissues, is a member of the bHLH-PAS (basic helix loop helix-period aryl hydrocarbon receptor nuclear translocator single-minded) family of transcription factors (4–7). *BMAL1* and its partner, CLOCK (circadian locomotor output cycles kaput), heterodimerize and bind to E-box elements in the promoter regions of clock-controlled genes to initiate their transcription, thereby driving the circadian rhythm (7).

A variety of physiological functions of *BMAL1* have been identified. Studies have revealed that *BMAL1-*knockout (KO) mice have multiple defects including premature aging (8, 9), skeletal dysplasia (10–12), abnormal hair growth (13, 14), and especially poor reproduction (15–17). *BMAL1-*KO mice were infertile (15–17), with defective gonads and gametes (15, 18–20). Moreover, the hypothalamus–pituitary–gonad (H-P-G) axis was found dysfunctional in these animals, including the abnormal secretion and signaling of gonadal steroids (19, 21, 22), gonadotropins (15, 23), and gonadotropin-releasing hormones (GnRH) (24). Also, *BMAL1-*KO mice exhibited problematic mating behaviors (25), uterine decidualization (26, 27) and embryo implantation (22). Moreover, accumulating clinical evidence has suggested that *BMAL1* also participates in human reproductive diseases (26–29), suggesting *BMAL1* as a potential theraputic target for related reproductive diseases.

## *BMAL1* in Reproductive Organ Development and Fertility

Various clues indicate that the development of the reproductive organs in *BMAL1-*KO mice is impaired (9, 16, 17, 22, 23, 30). Among such studies, these mice were reported to be arrhythmic under free-running conditions (constant darkness, constant temperature), with delayed puberty, irregular estrus cycles, and infertility (15, 16, 30). These fertility-impaired mice usually presented with structural and functional abnormalities in reproductive organs and gametes.

### Effect of *BMAL1* Knockout on Female Reproduction in Mice

Female *BMAL1-*KO mice displayed undersized and underdeveloped ovaries with a significant reduction in the number of corpora lutea and a higher proportion of atretic follicles (15). Despite these ovarian abnormalities, BMAL-KO female mice can ovulate; however, the quantity and quality of the oocytes are reduced (15, 16, 22, 31). A couple of studies reported that female *BMAL1-*KO mice had fewer ovulated oocytes and more abnormal oocytes after superovulation (15, 22, 32), which resembles the phenotypes of elder wild-type (WT) mice (15, 33), consistent with the premature aging feature of *BMAL1-*KO mice (9, 34). In addition, the *BMAL1-*KO mice exhibited abnormal follicle development, lower fertilization rate and retarded early embryo/blastocyst development (15, 32). Of note, although both *in vivo* fertilization in female *BMAL1-*KO mice and *in vitro* fertilization using *BMAL1-*KO oocytes were found to be impaired, the success rate of the *in vitro* fertilization was higher compared to that of *in vivo* fertilization in female *BMAL1-*KO mice (32), suggesting that the environment for fertilization and/or early embryo development might be affected by BMAL deletion. Further studies revealed high reactive oxygen species (ROS) levels in ovaries and oviducts of the female *BMAL1-*KO mice (32). Consistently, *BMAL1* was found as a direct regulator for ROS homeostasis (9, 34). Although ROS are important regulators of various physiological processes (35), excessive ROS could be detrimental for normal reproductive activities (36–38). Therefore, the excessive ROS in ovaries and oviducts may account for the defective follicular development, fertilization and early embryo development in female *BMAL1-*KO mice.

**Figure 1:**
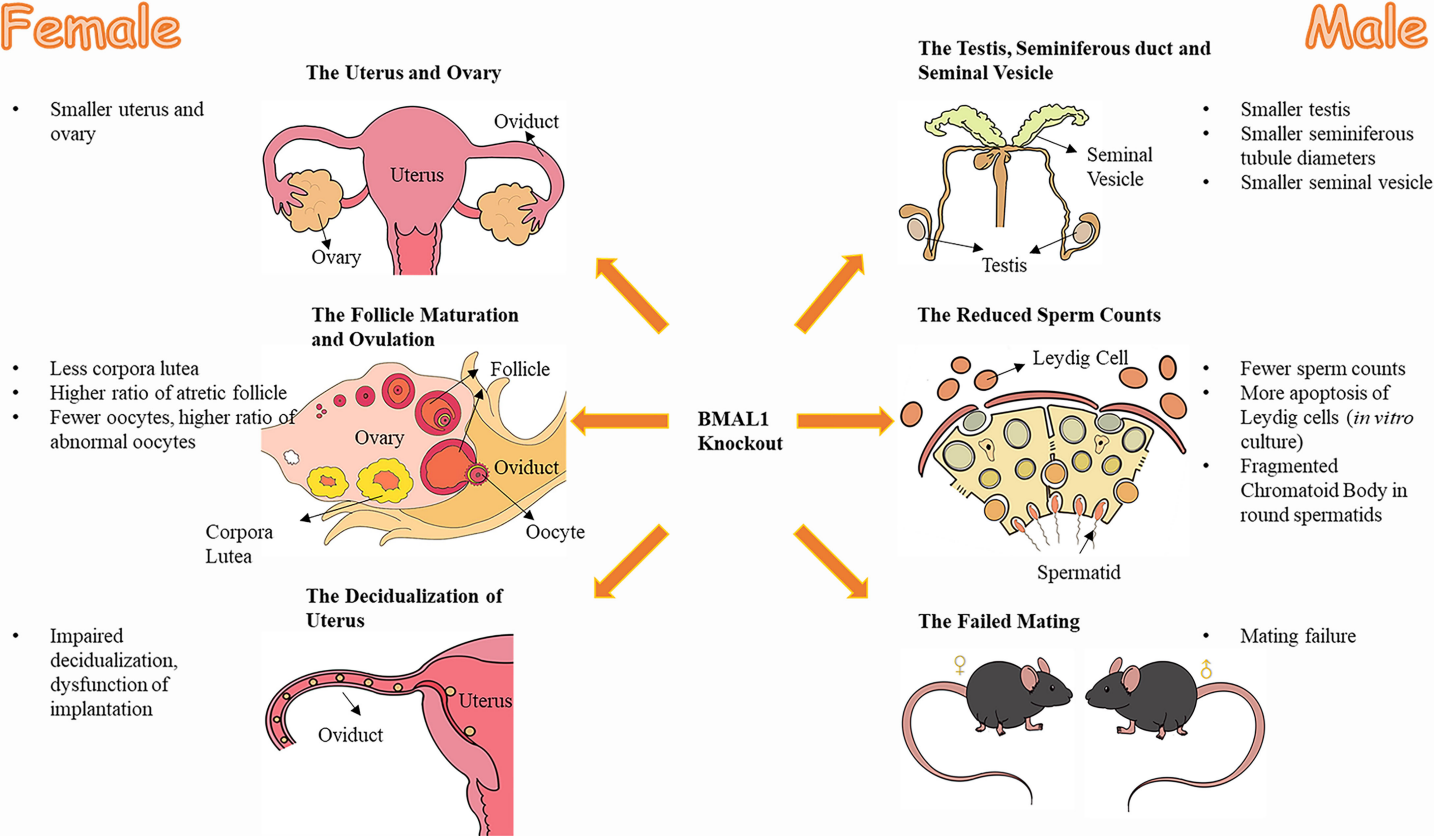
The effect of *BMAL1* knockout on mice reproduction. Both male and female *BMAL1-*KO mice suffered from varying degrees of dysplasia and dysfunction in their reproductive tissues. The female *BMAL1-*KO mice have smaller uteri and ovaries, with less corpora lutea, higher ratio of atretic follicles, fewer oocytes, higher ratio of abnormal oocytes, impaired decidualization procedures and thereby the impaired implantation function of the uterus. Male *BMAL1-*KO mice possess smaller testis, smaller seminiferous tubules, smaller seminal vesicles, with fewer sperms, more impaired Leydig cells, fragmented Chromatoid Body in round spermatids and their impaired behaviors of mating.

In addition, female *BMAL1-*KO mice had undersized uteri (15). Recent studies have revealed that *BMAL1* is associated with trophoblast invasion of the uterus decidualization (26, 27), which is a prerequisite for embryo implantation (39, 40). Furthermore, female *BMAL1-*KO mice were reported to experience an unsuccessful implantation (22, 32), since well-fertilized oocytes and well-developed preimplantation embryos could be observed in the reproductive tract on the implantation day (22). Therefore, the compromised implantation process of female *BMAL1-*KO mice can contribute to the impaired fertility of female *BMAL1-*KO mice (22). These structural abnormalities of the uterus and ovaries correlated with functional defects (**Figure 1**).

### Effect of *BMAL1* Knockout on Male Reproduction in Mice

Similar to the findings noted in females, male *BMAL1-*KO mice contained abnormal gonad and gametes. Male *BMAL1-*KO mice possessed slightly smaller testes, markedly smaller seminal vesicles, and generally reduced seminiferous tubule diameters (9, 23). The *in vitro* culture of TM3 Leydig cells have shown that silencing the *BMAL1* gene might contribute to the apoptosis of Leydig cells which produce testosterone (41).

Male *BMAL1-*KO mice exhibited a nearly 70% reduction in sperm count (23). However, other characteristics, including sperm motility, sperm capacitation, and the outcome of *in vitro* fertilization were normal, although there was a trend of slightly decreased successful fertilization (23). Consequently, the aforementioned findings, such as the decreased sperm counts and quality, could not serve as solid evidence to explain the infertility of male *BMAL1-*KO mice. Apart from that, *BMAL1* might also directly affect the structure of sperm. The chromatoid body (CB) is a special structure in pachytene spermatocytes and spermatids, which plays a pivotal role in gametogenesis during meiosis and spermatogenesis (42–44). Of interest, *BMAL1* depletion converted the CB into fragments in round spermatids (18), and *BMAL1* expression declined with abnormal CB structures and functions with age (45). As CB functions in mRNA silencing and translational regulation, which are important for spermatogenesis, *BMAL1* might interfere with such procedures in spermatogenesis (18). The abnormalities of the sperm might also contribute to the reduced fertility of *BMAL1-*KO mice. Furthermore, male *BMAL1-*KO mice failed to mate with receptive WT female mice, showing no mating behaviours (23, 25). The underlying mechanism will be outlined in the following section.

In conclusion, male *BMAL1-*KO mice exhibit defective gonads and abnormal mating behavior, factors that might account for male infertility (**Figure 1**).

## *BMAL1* in Hypothalamus-Pituitary-Gonad Regulatory Axis

Poor gonadal development and dysfunction usually indicate abnormal gonadotropins and GnRH levels. The secretion of gonadotropins, including luteinizing hormone (LH) and follicle-stimulating hormone (FSH), is controlled by upstream signals derived from the hypothalamus. There, GnRH is consistently recognized as the master regulator for the secretion of downstream gonadotropin, including FSH and LH derived from the pituitary (46–51). Additionally, another stimulator of gonadotropins, kisspeptin derived from Kiss1 neurons, especially in the anteroventral periventricular nucleus of the hypothalamus, is significantly associated with a GnRH surge and induces gonadotropins secretion; thus it could directly affect pituitary gonadotrophs to release LH and FSH (47, 49, 52–56).

### Effect of *BMAL1* Knockout on Hypothalamus/Pituitary Hormones in Mice

*BMAL1-*KO mice exhibited abnormal pituitary gonadotropin levels (15, 31, 57). In female mammals, ovulation occurs after a pro-estrous LH surge triggered by estradiol peaks (58). However, female *BMAL1-*KO mice did not present with an LH surge (57). Meanwhile, male *BMAL1-*KO mice exhibited significantly altered FSH levels both in serum and in the whole pituitary, with reduced mRNA expressions of FSHβ in the pituitary (23, 25). Thus, *BMAL1* absence could affect the secretion of gonadotropin from the pituitary.

To determine the reasons for abnormal gonadotropin levels, more studies have been conducted by focusing on the signaling mechanisms in the hypothalamus. GnRH secretion in *BMAL1-*KO mice was in normal range (57). In the meantime, Kiss1 neurons show normal c-Fos expression in Female *BMAL1-*KO mice at the time of the LH surge, suggesting a normal responding state of these neurons (57). Furthermore, female *BMAL1-*KO mice showed an enhanced LH response to exogenous kisspeptin and a normally induced LH response to exogenous GnRH, suggesting unimpaired reactivity in the pituitary of these female mice and an even higher sensitivity to exogenous kisspeptin (57). Similarly, male *BMAL1-*KO mice also exhibited higher sensitivity to kisspeptin and GnRH, with normal expression of GnRH and Kisspeptin in the hypothalamus (25). Notably, the GnRH receptor (GnRHR) gene was established to contain an E-box in the promoter region, and was under the regulation of *BMAL1* (59). In Female *BMAL1-*KO mice, the expression of *GnRHR* in the pituitary remained unchanged, but that in Male *BMAL1-*KO mice was reduced (25). Therefore, *BMAL1* participates in signaling from the hypothalamus to the pituitary. However, in terms of the lost LH surge and the abnormal reactivity of the pituitary to the hypothalamus in *BMAL1-*KO mice, there might be some additional mechanisms underlying the abnormal signaling from the hypothalamus to the pituitary.

Deleting *BMAL1* specifically in important cells associated with the GnRH/LH pathways also resulted in alterations in gonadotropin release and response patterns (60). Conditional *BMAL1* knockout in arginine vasopressin (AVP) neurons, which were demonstrated to play a specific role in the LH surge, resulted in a low and delayed LH surge; In KISS1 neuron conditional *BMAL1-*KO mice, the secretion pattern with double peaks and an altered surge time of induced LH were noted. Furthermore, in GnRH-neuron conditional *BMAL1-*KO mice, the LH surge was noted disappeared at a specific time, and the amplitude was found to be low (60). Such cells played important roles in hypothalamus endocrine signal transmission and were also proved to participate in mechanisms involving in GnRH/LH signaling (60). Therefore, the altered timing and pattern of the LH surge in these mice indicated that *BMAL1* in these cells was involved in the transmission and feedback of hormones from the hypothalamus to the pituitary.

Altogether, *BMAL1* participates in hormone secretion and signaling of hypothalamus and pituitary.

### Effect of *BMAL1* Knockout on Hypothalamus-Pituitary Regulation of Reproduction in Mice

LH and FSH are considered pivotal signals for gonadal development, follicle development, spermatogenesis, gonadal steroidogenesis, constitute the key steps for reproductive outcomes (61–67).

Female *BMAL1-*KO mice did not have an LH surge, an important stimulus for periodic ovulation. It is possible that the impaired quantity and quality of the superovulated oocytes of female *BMAL1-*KO mice were associated with the phenomenon (15). Apart from ovulation, LH also plays a significant role in the maintenance of corpora lutea (68, 69). This was consistent with the reduced number of corpora lutea and the disordered structure of the ovaries (15). Moreover, the delayed puberty and the disordered estrous cycle might also be attributed to the disrupted production of hormone signals (15). Such abnormal hypothalamus and pituitary hormone secretion and signaling in female *BMAL1-*KO mice might be associated with the impaired fertility.

Male *BMAL1-*KO mice had higher levels of LH, a hormone that primarily functions through Leydig cells to produce testosterone for male reproduction. Thus, the higher LH level in male *BMAL1-*KO mice was in line with the comparatively low testosterone levels. FSH facilitates male reproduction by promoting the proliferation of Sertoli cells and spermatogonia, thereby increasing sperm counts in synergy with testosterone (70–72). Therefore, it is possible that the lower levels of FSH and testosterone in male *BMAL1-*KO mice were responsible for the decreased sperm counts. Moreover, male *BMAL1-*KO mice showed reduced hypothalamic expressions of vasoactive intestinal peptide (Vip) and tyrosine hydroxylase (Th) (25), both of which are associated with the mating behaviors of male mice (73–75). The abnormal hypothalamus and pituitary hormone secretion and signaling of male *BMAL1-*KO mice were possibly associated with low sperm counts and impaired mating behaviors in these mice.

Altogether, *BMAL1* may affect hormone secretion and signaling of hypothalamus and pituitary, thus affecting the fertility of *BMAL1-*KO mice.

## *BMAL1* in Gonadal Hormone Production

Reproductive activities are usually associated with gonadal hormones. Indeed, abnormal gonadal hormone levels might directly result in severe defects in the reproduction of *BMAL1-*KO mice.

### Effect of *BMAL1* Deficiency on Gonadal Hormone Secretion and Actions

In females, progesterone and estradiol are considered crucial hormones for embryo implantation and maintenance (39). However, *BMAL1* depletion impaired female gonadal hormone secretion. Indeed, female *BMAL1-*KO mice showed decreased progesterone levels during the estrus cycle and early gestation (15, 22, 76). *BMAL1* knockdown by small interfering RNA (siRNA) in *in vitro* cultured porcine granulosa cells and luteinizing granulosa cells of rat and human, also led to a decrease in progesterone and estradiol synthesis (21, 77, 78). Additionally, evidence suggests that leptin and leptin receptor (*Lepr*) exerted positive effects on estradiol synthesis in murine granulosa cells, whereas *BMAL1* knockdown reduced estradiol synthesis and suppressed the stimulating effect of leptin and *lepr* on estradiol synthesis and associated gene expressions (20). Therefore, *BMAL1* plays a role in the process by which leptin signaling induces estradiol synthesis (20). Another hormone, prostaglandin E2 (PGE2), was also found to be reduced in the reproductive tissues of female *BMAL1-*KO mice (21, 79). Regulated by rate-limiting enzymes encoded by the gene prostaglandin-endoperoxide synthase 2/cyclooxygenase 2 (ptgs2 or cox2), PGE2 has been deemed vital for ovulatory cascade, sperm penetration, and fertilization, and decidual preparation for implantation (80–84). Moreover, *in vitro* cultured *BMAL1* rat luteinizing granulosa cells and *BMAL1-*KO uterus cells exhibited considerably downregulated ptgs2 expression, and PGE2 secretion (21, 79). Therefore, *BMAL1* is important for female gonadal hormone secretion, which could directly account for the impaired fertility of female *BMAL1-*KO mice. Female *BMAL1-*KO mice were reported to undergo unsuccessful implantation. Exogenous supplementation with progesterone substantially rescued the implantation failure, although the recovered implantation sites were smaller and fewer compared to those noted in the WT group (22). Similarly, in female steroidogenic factor-1 (SF1) conditional *BMAL1-*KO mice, also characterized by poor steroidogenesis as well as failed implantation, exogenous progesterone supplement could greatly improve the implantation rate (76). Furthermore, progesterone was found to participate in the uterus decidualization pathways (39, 84–86), which are influenced by *BMAL1* depletion (27). Thus, the impaired steroidogenesis due to *BMAL1* loss might underlie the failed implantation of *BMAL1-*KO mice.

In males, testosterone also plays a crucial role in testis development and spermatogenesis (87–89). In line with the morphological abnormalities of the testis and seminal vesicles, male *BMAL1-*KO mice showed reduced testosterone secretion (23, 25, 90). In fact, *BMAL1* was reported to be only expressed and oscillate in Leydig cells which account for testosterone production (19, 23), whereas *BMAL1* oscillation in the whole testis was demonstrated to be arrhythmic (91, 92). In goat Leydig cells and *in vitro* cultured TM3 Leydig cells, *BMAL1* knockdown led to a reduction in testosterone levels (41, 93). Thus, *BMAL1* loss could be responsible for the poor gonadal hormone secretion in male *BMAL1-*KO mice.

Overall, the existing evidence for implantation related infertility in *BMAL1-*KO mice can be considered closely associated with the abnormal secretion of reproductive hormones.

### Effect of *BMAL1* Deficiency on Gonadal Hormone Genes Expression

In line with the impaired steroidogenesis, *BMAL1-*KO and interference altered the expression of most steroidogenic genes, including steroidogenic acute regulatory protein (*StAR*), cytochrome P450 family 11 subfamily A member 1(*Cyp11a1*), 3β-hydroxysteroid dehydrogenase 2 (Hsd3b2), 17-β-hydroxysteroid dehydrogenase 3 (Hsd17B3), cytochrome P450 family 17 subfamily A member 1(*Cyp17a1*), and cytochrome P450 aromatase (Cyp19a1). *BMAL1-*KO mice also altered the expression of related hormone receptor genes, including follicle-stimulating hormone receptor (*FSHr*), luteinizing hormone receptor (*LHr, Lhcgr*), and estrogen receptor β (*ERβ*) (15, 20–23, 41, 77, 94, 95) (**Table 1**). For example, *BMAL1* knockdownin goat Leydig cells reduced testosterone production and the expression of *StAR* and HSD3B2, whereas *BMAL1* overexpression enhanced testosterone production and the expression of *StAR* and Hsd17b2 (93). In rat luteinizing granulosa cells, *BMAL1* knockdown suppressed the expression of a series of ovarian genes, such as *StAR*, *Cyp19a1*, *Cyp11a1*, *Ptgs2*, *Lhcgr*, and *Hsd3b2*, with reduced progesterone and PGE2.

**Table 1:** The gonadal steroidogenic genes and their association with circadian clock.

Reference	Gene	E-box	Rhythmic
(19, 21, 23, 77, 93, 95–97)	*StAR* (Steroidogenic acute regulatory protein)	Yes	Yes
(19, 21, 77, 93)	*Cyp11a1* (Cytochrome P450 family 11 subfamily A member1, Cytochrome P450 cholesterol side chain cleavage)	Yes	Yes
(19, 93, 95)	*Cyp17a1* (Cytochrome P450 family 17 subfamily A member 1, Cytochrome P450 17alpha-hydroxylase/17,20-lyase)	–	Yes
(21, 77, 95)	*Cyp19a1* (Cytochrome P450 family 19 subfamily A member 1, Cytochrome P450 aromatase)	Yes	Yes
(21, 93, 95)	*Hsd3b2* (3β-Hydroxy-Δ5-steroid dehydrogenase)	No	No
(19)	*Hsd17b3* (3β‐hydroxysteroid dehydrogenase)	Yes	No
(21, 98)	*Pgst2* (Prostaglandin-endoperoxide synthase 2/cyclooxygenase 2)	Yes	Yes
(77, 99, 100)	*ER(β)* (Estradiol Receptor β)	Yes	Yes
(77)	*FSHr* (Follicle-stimulating hormone receptor)	No	Yes
(21, 77, 95)	*LHr* (Lhcgr, Luteinizing hormone receptor)	No	Yes
(20)	*Lepr* (LeptinR, Leptin Receptor)	Yes	Yes
(59)	*GnRHR* (Gonadotropin-Releasing-Hormone Receptor)	Yes	Yes

Among steroidogenic genes, *StAR* is necessary for cholesterol transfer into the mitochondrial membrane (101). Both male and female *BMAL1-*KO mice reduced *StAR* protein and mRNA expression both *in vivo* and *in vitro* (15, 21–23, 41). Moreover, the *StAR* gene contains an E-box element near its promoter region, and *BMAL1* can directly induce its transcription (23, 96, 97). Similarly, the expression of *HSD17B3* was also demonstrated to be directly regulated by *BMAL1* (93). *Erβ*, a major estradiol receptor type in the SCN, plays a role in reproduction, especially in follicle maturation (102–105). With an E-box in its promoter, *BMAL1* was demonstrated to directly regulate Erβ expression (99). *Lepr* also contains an E-box in its promoter and is subjected to regulation by *BMAL1* (20). In summary, *BMAL1* can affect steroidogenesis and hormone signaling by directly affecting certain steroidogenesis related genes.

### Effect of *BMAL1* Deficiency on Signaling Pathways Associated With Steroidogenesis

Silencing information regulator 2 related enzyme 1 (sirtuin1, SIRT1) is an NAD^+^-dependent deacetylase; it collaborates with *BMAL1* in the circadian regulation (106–109). It has already been established that SIRT1 participates in the steroidogenic function of estrogen-producing cells. Further evidence confirmed that SIRT1-*BMAL1* signaling is involved in estradiol production in steroidogenic cells, by regulating the c-Jun N-terminal kinase (JNK) pathway (78). Moreover, *BMAL1*, SIRT1, and the JNK pathways seem to mutually affect each other, forming a *BMAL1-*SIRT1-JNK loop that functions in gonadal steroidogenesis (78).

Studies have revealed that the PI3K/AKT/mTOR pathway participates in the biological processes of apoptosis and steroidogenesis in granulosa and Leydig cells (110–114). Coincidently, in the *in vitro* cell culture of porcine granulosa cells and the TM3 Leydig cell line, the PI3K/AKT/mTOR pathway was suppressed after intervention of BMAL1,with reduced steroidogenesis and increased apoptosis of steroidogenic cells (41, 77). Notably, this pathway was also reported to be involved in leptin-*lepr* signaling in the regulation of steroidogenesis (20). Therefore, *BMAL1* might have functions in the PI3K/AKT/mTOR pathway and thus play a role in the apoptosis of gonadal steroid hormone-producing cells and steroidogenesis.

The PI3K/NF-κB pathway is also involved in steroidogenesis. NF-κB was demonstrated to participate in circadian rhythm generation and was also associated with the PI3K/AKT/mTORC1 pathway, which functions in steroid hormone production (110–114). Female *BMAL1-*KO mice exhibited impaired steroidogenesis and activated phosphorylation of the PI3K/NF-κB pathway. Moreover, data from the *in vitro* cultured mice theca and granulosa cells confirmed that the *BMAL1* knockdown increased phosphorylation of the PI3K/NF-κB pathway, along with severely impaired luteal steroidogenesis (95). Further evidence indicated that *BMAL1* might directly establish negative interactions with NF-κB p (RelA), a subunit of NF-κB (95). Therefore, *BMAL1* is also involved in the regulation of PI3K/NF-κB pathway, inducing steroidogenesis.

## *BMAL1* in the Etiology of Human Reproductive Pathogenesis

Accumulating clinical evidence has demonstrated the role of *BMAL1* in human reproductive pathologies. Single nucleotide polymorphisms (SNPs) of circadian genes revealed that *BMAL1* is associated with all epithelial ovarian cancers (EOCs) and their histopathologic subtypes (28). Moreover, *BMAL1* was obviously downregulated in an early stage transformed model of EOCs (28). One variant of *BMAL1* was previously linked to a higher number of pregnancies and a higher number of miscarriages (29). In addition, *BMAL1* expression was downregulated in recurrent miscarriage patients (26), and *BMAL1* was revealed to affect trophoblast invasion during decidualization by regulating TIMP3, a protein that controls the invasiveness of trophoblast cells (26). Another mechanistic study revealed that *BMAL1* facilitated the migration and invasion of the extra-villous trophoblast (EVT) *via* the SP1-DNMT1/DAB2IP pathway, which could be the underlying mechanism through which progesterone treatment prevents spontaneous abortion (27).

## Conclusions and Prospects

As current review has indicated, *BMAL1* knockout leads to impaired structures and functions of reproductive organs and gametes, signaling from the hypothalamus to pituitary impaired gonadal hormone secretion and impaired hormone secretion. Furthermore, dysfunctional *BMAL1* was demonstrated to participate in human reproductive diseases. These findings revealed the critical role of *BMAL1* in reproductive endocrinology and fertility. Still, the exact underlying mechanisms of how *BMAL1* impacts on reproduction need to be further explored. The disparate phenotypes in different *BMAL1-*KO models (**Table 2**) have offered us deep insights into the role of *BMAL1* in reproduction. Among these mice, global *BMAL1-*KO mice and steroidogenesis cells conditional *BMAL1-*KO (SF1- KO) mice were infertile, while other conditional *BMAL1-*KO mouse models were fertile such as the induced *BMAL1-*KO (iKO) mice (115). The iKO mice were treated with tamoxifen (TAM) at 8-weeks of age, thus losing *BMAL1* in adulthood, and in these animals, reproductive tissues and reproductive endocrine systems are well-developed while devoid of BMAL1. However, *BMAL1-*KO mice had impaired reproductive tissues and gametes, as well as impaired reproductive endocrine systems. Thus, *BMAL1* has a critical role in reproductive endocrinology and fertility.

**Table 2:** Different *BMAL1-*KO mice and the varied reproductive phenotypes.

Reference	Knockout Mice	The Reproductive Phenotypes
(15, 22, 31, 115)	Global *BMAL1-*KO (*BMAL1-*KO)	Infertile in both female and male mice; delayed puberty in both sexes; prolonged progression through the estrous cycle in females
(116)	Myometrium-*BMAL1-*KO	Fertile; altered parturition time
(31)	Pituitary Gonadotrope-*BMAL1-*KO (G*BMAL1-*KO)	Fertile; successful and viable offspring; irregular estrous cycle
(76)	Steroidogenesis cells *BMAL1-*KO (SF1-*BMAL1-*KO)	Infertile; normal puberty; signs of early pregnancy loss and re-entry into estrus after mating
(95, 117)	Ovarian Theca Cell *BMAL1-*KO (TCKO)	Fertile; more mating failure with less viable litters; normal estrous cycle
(95, 117)	Ovarian Granular cell *BMAL1-*KO (GCKO)	Fertile; normal estrous cycle; normal ovary morphology
(115)	Inducible *BMAL1* KO (iKO)	Fertile; less fertile than normal mice, but comparable to the controls

As the core transcription factor of the circadian rhythm, *BMAL1* also serves its circadian function in regulating the rhythm of reproductive endocrine hormones. Indeed, reproductive endocrine hormones like estradiol (20), and testosterone (118, 119), have been demonstrated to be rhythmically released. And the associated steroidogenic genes showed robust circadian rhythm in corresponding steroidogenic cells (20, 90, 93). Moreover, the disruption of *BMAL1* by siRNA in mice steroidogenic cells led to the disrupted rhythm of associated genes including *StAR*, *Cyp11a1*, Hsd3b2, and *Hsd17b3* (20, 90, 93). Additionally, another gene family crucial for cholesterol transportation to produce testosterone in the testis, namely apolipoprotein (Apo) genes (including Apoa1, Apoa2 and Apoc3), was reported to be expressed in a robust circadian manner in WT mice (90). In *BMAL1-*KO mice, the rhythmicity of the expressions of Apo genes is lost, along with impaired metabolism of cholesterol, which might be associated with the reduced testosterone in Male *BMAL1-*KO mice (90). Accordingly, the circadian gene *BMAL1* can also influence reproductive endocrinology by regulating the rhythmic expression of corresponding genes.

In conclusion, the established mouse model and the mechanistic studies have provided insights into the role of *BMAL1* in reproduction. This could have clinical significance, as more reproductive pathologies can be attributed to defects at the gene level, such as the key role of *BMAL1* outlined in this review. It is anticipated that a deeper understanding of the role of *BMAL1* in reproduction could be applied to clinical practices in reproductive disease pathology and target therapies in the future.

## Author Contributions

DM and JL devised topic. YJ and SL wrote the draft. JY and YJ devised tables. AZ, YY, RZ, and TR devised figures. WX, YQ, and XJ revised for accuracy. DM financially support this work. All authors contributed to the article and approved the submitted version.

## Funding

This study was partially supported by National Natural Science Foundation of China (81971433, 81701500); the grants from the Science and Technology Bureau of Sichuan Province (2021YJ0017, 2020YFS0041).

## Conflict of Interest

The authors declare that the research was conducted in the absence of any commercial or financial relationships that could be construed as a potential conflict of interest.

## Publisher’s Note

All claims expressed in this article are solely those of the authors and do not necessarily represent those of their affiliated organizations, or those of the publisher, the editors and the reviewers. Any product that may be evaluated in this article, or claim that may be made by its manufacturer, is not guaranteed or endorsed by the publisher.
